# Supplementary material for: 1H NMR Profiling of Honey Bee Bodies Revealed Metabolic Differences between Summer and Winter Bees
Source: Insects. 2022 Feb 12;13(2):193. doi: 10.3390/insects13020193 (PMC8875373; doi:10.3390/insects13020193)
Supplement: Supplementary file 1 [file insects-13-00193-s001.zip › insects-1533397-supplementary.pdf]

insects

MDPI

Article

1H NMR Profiling of Honey Bee Bodies Revealed Metabolic Differences between Summer and Winter Bees

Saetbyeol Lee [1], Filip Kalcic [1] ,Iola F. Duarte [2], Dalibor Titera [3], Martin Kamler [4], Pavel Mrna [1], Pavel Hyrsl [5], Jiri Danihilik [6], Pavel Dobes [5], Martin Kunc [5], Anna Pudlo [1], and Jaroslav Havlik [1,*]

1 Department of Food Science, Faculty of Agrobiology, Food and Natural Resources, Czech University of Life Sciences Prague, Kamycka 129, 165 00, Prague, Czech Republic; lees@af.czu.cz (S.L.); filip.kalcic@gmail.com (F.K.); mrna.pavel@gmail.com (M.P.); anna.pudlo@upwr.edu.pl (A.P.)

2 University of Aveiro (CICECO), Campus de Santiago, 3810-19, Aveiro, Portugal; ioladuarte@ua.pt

3 Department of Zoology and Fisheries, Faculty of Agrobiology, Food and Natural Resources, Czech University of Life Sciences Prague, Kamycka 129, 165 00, Prague, Czech Republic; titera@af.czu.cz

4 Honeybee Research Institute, Dol 94, 252 66, Maslovice, Czech Republic; martin.kamler@gmail.com

5 Department of Experimental Biology, Faculty of Science, Masaryk University, Kamenice 5, 625 00, Brno, Czech Republic; hyrsl@sci.muni.cz (P.H.); pavel.dobes@mail.muni.cz (P.D.); sikfrid.kunc@gmail.com (M.K.)

6 Department of Biochemistry, Faculty of Science, Palacky University Olomouc, Slechtitelu 27, 783 71, Olomouc, Czech Republic; jiri.danihilik@upol.cz

* Correspondence: havlik@af.czu.cz; Tel.: +420

Citation: Lee, S.; Kalcic, F.; Duarte, I.F.; Titera, D.; Kamler, M.; Mrna, P.; Hyrsl, P.; Danihlik, J.; Dobes, P.; Kunc, M.; et al. 1H NMR Profiling of Honey Bee Bodies Revealed Metabolic Differences between Summer and Winter Bees. *Insects* **2022**, *13*, 193. https://doi.org/10.3390/insects13020193

Academic Editors: Guy Bloch and Christian Wegener

Received: 15 December 2021
Accepted: 8 February 2022
Published: 12 February 2022

Publisher's Note: MDPI stays neutral with regard to jurisdictional claims in published maps and institu-tional affiliations.

Copyright: © 2022 by the authors. Submitted for possible open access publication under the terms and conditions of the Creative Commons Attribution (CC BY) license (https://creativecommons.org/licenses/by/4.0/).

Supplementary Data

Table S1. Contributions of the variables to the principal components in percentage (%)

	PC1	PC2
**AMP**	2.58	0.33
**Acetate**	0.29	0.26
**Adenine**	4.93	0.54
**Adenosine**	4.16	5.42
**Alanine**	0.16	3.00
**Asparagine**	3.01	0.35
**Choline**	3.02	0.75
**Creatine**	0.69	0.22
**Fructose**	5.05	23.15
**Glucose**	1.49	17.94
**Glutamine**	0.04	1.29
**Glycine**	0.35	3.12
**Histidine**	0.18	0.26
**Inosine**	1.70	0.00
**Isoleucine**	2.55	0.00
**Leucine**	6.31	0.80
**Lysine**	2.35	0.66
**Malonate**	0.12	2.07

**NAD⁺**	4.84	1.68
***O*-Phosphocholine**	2.06	1.54
**Phenylalanine**	7.16	0.89
**Proline**	1.20	0.04
**Putrescine**	0.25	0.14
**Sarcosine**	1.46	1.91
**Suberate**	0.02	4.46
**Succinate**	1.90	0.01
**Sucrose**	10.74	20.69
**Taurine**	0.14	0.00
**Threonine**	1.72	3.52
**Trehalose**	5.89	0.28
**Trimethylamine N-oxide**	6.88	1.36
**Unknown**	11.59	1.34
**Uridine**	0.22	0.00
**Valine**	4.72	0.05
***sn*-Glycero-3-phosphocho**	0.01	0.45
**β -Alanine**	0.21	1.47

22
